# JAK inhibitor has the amelioration effect in lupus-prone mice: the involvement of IFN signature gene downregulation

**DOI:** 10.1186/s12865-017-0225-9

**Published:** 2017-08-22

**Authors:** Keigo Ikeda, Kunihiro Hayakawa, Maki Fujishiro, Mikiko Kawasaki, Takuya Hirai, Hiroshi Tsushima, Tomoko Miyashita, Satoshi Suzuki, Shinji Morimoto, Naoto Tamura, Kenji Takamori, Hideoki Ogawa, Iwao Sekigawa

**Affiliations:** 1 Department of Internal Medicine and Rheumatology, Juntendo University Urayasu Hospital, 2-1-1 Tomioka Urayasu-shi, Chiba, 279-0021 Japan; 2 Institutes for Environmental and Gender Specific Medicine, Juntendo University Graduate School of Medicine, Chiba, Japan; 3 Department of Internal Medicine and Rheumatology, Juntendo University School of Medicine, Tokyo, Japan

**Keywords:** Systemic lupus erythematosus, Interferon, Cytokines, T cells, JAK–STAT pathway, Translational research

## Abstract

**Background:**

We previously reported that JAK–STAT-pathway mediated regulation of IFN-regulatory factor genes could play an important role in SLE pathogenesis. Here, we evaluated the efficacy of the JAK inhibitor tofacitinib (TOFA) for controlling IFN signalling via the JAK–STAT pathway and as a therapeutic for SLE.

**Results:**

We treated NZB/NZW F1 mice with TOFA and assessed alterations in their disease, pathological, and immunological conditions. Gene-expression results obtained from CD4^+^ T cells (SLE mice) and CD3^+^ T cells (human SLE patients) were measured by DNA microarray and qRT-PCR. TOFA treatment resulted in reduced levels of anti-dsDNA antibodies, decreased proteinuria, and amelioration of nephritis as compared with those observed in control animals. Moreover, we observed the rebalance in the populations of naïve CD4^+^ T cells and effector/memory cells in TOFA-treated mice; however, treatment with a combination of TOFA and dexamethasone (DEXA) elicited a stronger inhibitory effect toward the effector/memory cells than did TOFA or DEXA monotherapy. We also detected decreased expression of several IFN-signature genes *Ifit3* and *Isg15* in CD4^+^ from SLE-prone mice following TOFA and DEXA treatment, and *IFIT3* in CD3^+^ T cells from human patients following immunosuppressant therapy including steroid, respectively.

**Conclusion:**

Modulation of type I IFN signalling via JAK–STAT inhibition may exert a beneficial effect in SLE patients, and our results suggest that TOFA could be utilised for the development of new SLE-specific therapeutic strategies.

**Electronic supplementary material:**

The online version of this article (doi:10.1186/s12865-017-0225-9) contains supplementary material, which is available to authorized users.

## Background

SLE is a chronic inflammatory disease that affects multiple organs and is a representative autoimmune disease in humans. It is characterised by the activation of dendritic cells, macrophage and lymphocytes, which results in the production of several high-affinity autoantibodies and formation of immune complexes. Notably, despite numerous studies conducted in human patients and mouse models, details concerning SLE pathogenesis remain unclear; however, a variety of genes [1] and environmental factors, including viral infections, hormones [2, 3], and ultraviolet rays, have been suggested as exacerbation factors to SLE development.

Multiple cytokines, including type I IFN [4, 5], type II IFN-γ [6], IL-2, and IL-6, play key roles in SLE initiation, progression, and development. Notably, each of these cytokines transmits signals via receptors controlled by the Janus kinase–signal transducer and activator of transcription (JAK–STAT)-signalling pathway [7]. Previously, we analysed differences in the gene-expression profiles of peripheral blood CD3^+^ T cells obtained from patients in the active / inactive phases of SLE. Our findings suggested that JAK–STAT-pathway mediated regulation of IFN-regulatory factor (IRF)-related genes might contribute to SLE pathogenesis and disease activity [8]. Recently, the JAK inhibitor tofacitinib (TOFA), which inhibits the JAK family of protein tyrosine kinases, was utilised as an immunosuppressive and anti-inflammatory agent for the treatment and prevention of acute allograft rejection [9], rheumatoid arthritis (RA) [10, 11], and psoriasis [12]. Moreover, single-targeted therapies [e.g., cytotoxic T-lymphocyte-associated protein 4-Ig, IL-6-receptor inhibitors, and CD20 inhibitors] have been remarkably effective for treatment of RA; however, these therapies fail to achieve satisfactory outcomes in SLE patients [13, 14]. Therefore, based on our previous findings regarding the role of IFN in SLE via the JAK–STAT pathway and the fact that TOFA comprises a multi-targeted therapeutic, we evaluated the efficacy of TOFA as a novel SLE-treatment option.

## Results

### TOFA administration reduces levels of anti-dsDNA antibodies, decreases proteinuria, and improves splenomegaly in SLE-prone mice

**Fig. 1:**
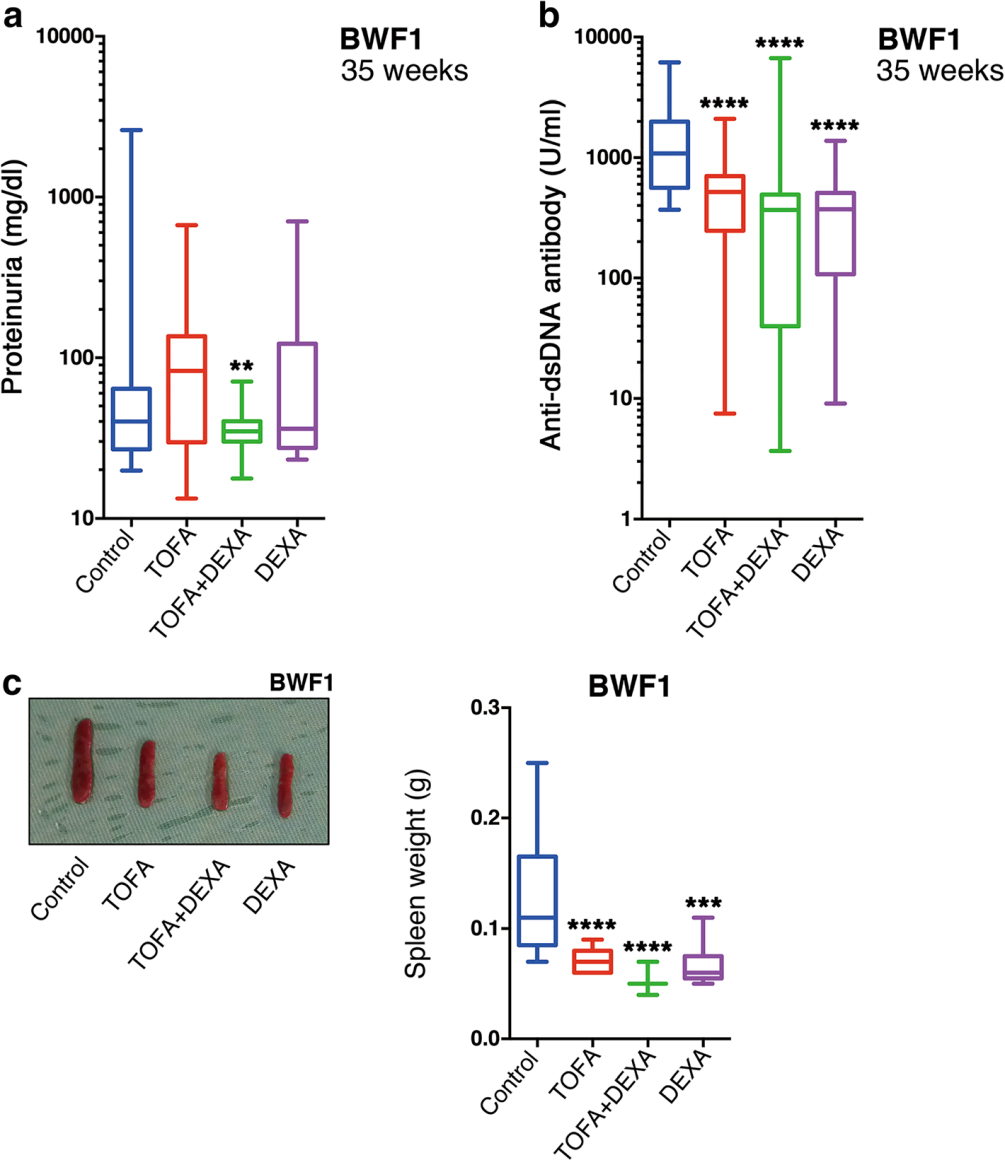
TOFA improved clinical manifestations in BWF1 mice. Urine and blood were evaluated for (**a**) proteinuria and (**b**) anti-dsDNA antibody titres in BWF1. Spleen size (**c**, left) and weight in BWF1 (**c**, right). Boxplot shows median and interquartile ranges from minimum to maximum [TOFA (BWF1: *n* = 13); TOFA + DEXA (BWF1: *n* = 15); DEXA (BWF1: *n* = 8); and control (BWF1: *n* = 13)]. **p < 0.01; ***p < 0.001 and ****p < 0.0001 (vs. control mice). BWF1: NZB/NZW F1 mice; DEXA: dexamethasone; TOFA: tofacitinib

To evaluate the efficacy of TOFA treatment against SLE, we used autochthonous SLE-model, BWF1 [15] mice. For these analyses, we administered TOFA alone or in combination with 0.5 mg/kg DEXA to each mouse. Notably, each of the TOFA + DEXA-treated groups exhibited significantly decreased proteinuria (Fig. 1a) and reduced anti-dsDNA-antibody titres (Fig. 1b), which are indicators of SLE activity, as compared with levels observed in control animals.

TOFA-treated and control mice were sacrificed and dissected following discontinuation of treatment. The spleens of TOFA-treated mice were clearly smaller than those of control mice (Fig. 1c). Similarly, the spleens of BWF1 in the TOFA-treatment groups weighed significantly less than those of control mice. In particular, dual therapy with TOFA + DEXA resulted in the greatest degree of reduction in spleen weight of BWF1 mice (Fig. 1c). Additionally, most strongly significant decreases of spleen size and weight were also observed in different SLE-model MRL mice treated with TOFA + DEXA (Additional file 1: Figure S1a).

### TOFA treatment ameliorates nephritis in SLE-prone mice

**Fig. 2:**
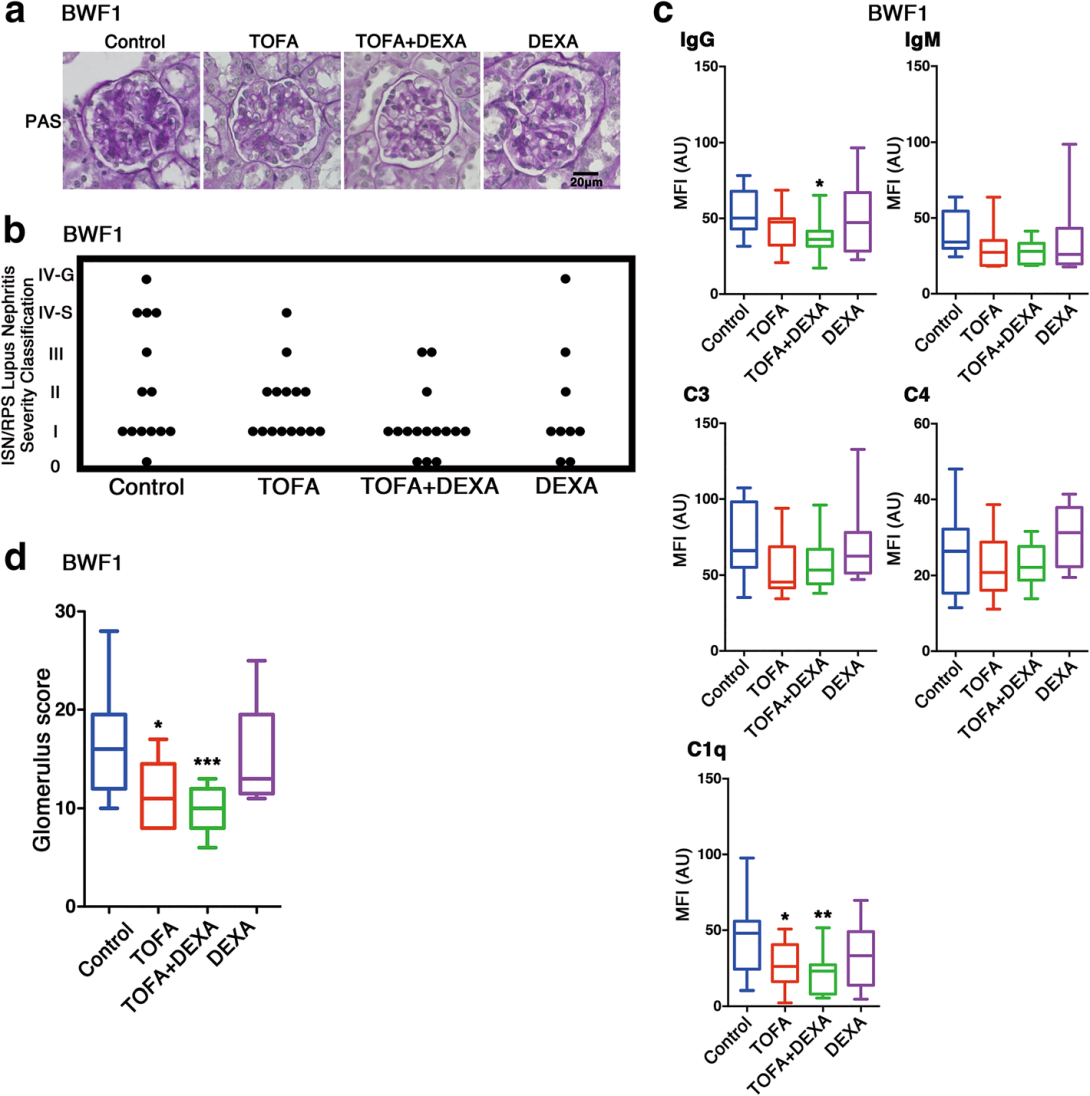
TOFA ameliorated glomerular nephritis in BWF1. Evaluation of kidneys from BWF1 for glomerular nephritis. (**a**) Representative PAS stained kidney sections. (**b**) Evaluation of nephritis severity. (**c**) MFI values of immunofluorescence staining in kidney sections. (**d**) Glomerulus score in control and each treatment group. Each represents [TOFA (*n* = 13); TOFA + DEXA; (*n* = 15); DEXA (*n* = 8); and control (*n* = 13)]. *p < 0.05; **p < 0.01; and ***p < 0.001 (vs. control mice)

SLE-prone mice develop renal dysfunction. Notably, BWF1 mice tend to specifically develop glomerular nephritis [15]. To evaluate the effects of TOFA treatment on these conditions, kidneys were harvested from mice in each treatment group, sectioned, and subjected to microscopic analysis. The severity of glomerular nephritis in BWF1 mice was evaluated based on the classification system of the ISN/RPS [16, 17]. While the kidneys of untreated BWF1 mice presented with nephritis with mesangial accumulation and hypercellularity, those in the TOFA-monotherapy and TOFA + DEXA dual-therapy groups exhibited normal glomeruli and a decreased frequency of severe-stage glomerular nephritis (Fig. 2a and b). The immunofluorescence intensities of IgG and C1q were significantly lower in tissues harvested from BWF1 mice in the TOFA + DEXA-treated groups as compared with those in tissues of control mice (Fig. 2c). To analyse the severity of glomerular nephritis statistically, we configured the glomerulus score, the criterion to decide the severity. The glomerulus score based on the MFI of the immunofluorescence and the classification of ISN/RPS was used for the severity of glomerular nephritis (Additional file 2: Table S1). The TOFA and TOFA + DEXA therapy groups showed significantly lower glomerulus scores as compared with the control group (Fig. 2d). Interstitial nephritis in MRL mice was evaluated by measuring the levels of cell infiltration via the analysis of microscopic images. Similarly, most strongly significant reductions in the levels of cell infiltration were observed in MRL mice treated with TOFA + DEXA (Additional file 1: Figure S1b and c).

### TOFA treatment decreases T cell activity

**Fig. 3:**
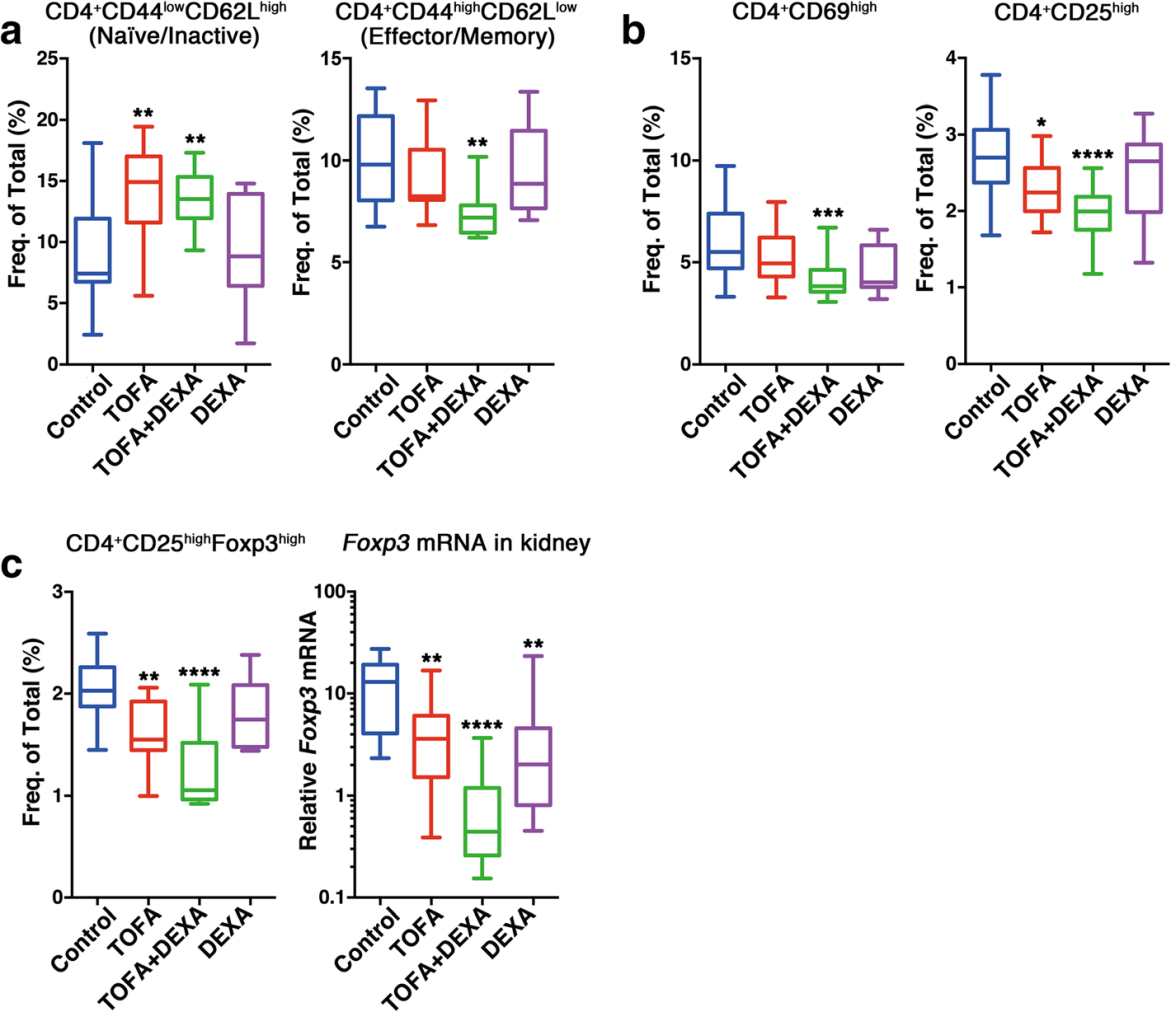
TOFA rebalanced immunocytes in BWF1. (**a**) Percentages of naïve/inactive T or T_EM_ cells from each mouse group. (**b**) Percentages of CD4^+^CD69^high^ or CD4^+^CD25^high^ T cells from each mouse group. (**c**, left) Percentages of T_reg_ cells from each mouse group. (**c**, right) *Foxp3* expression in kidneys of BWF1. Each represents [TOFA (*n* = 13–15); TOFA + DEXA (*n* = 15); DEXA (*n* = 8); and control (*n* = 13–14)]. *p < 0.05, **p < 0.01, ***p < 0.001 and ****p < 0.0001 (vs. control mice). Freq. of Total (%): frequency of total splenocytes; T_EM_: effector/memory T; T_reg_: regulatory T

Previous studies detected abnormalities in the function, distribution, and gene-expression patterns of various T cell subsets in both SLE patients and in lupus-prone mouse model [18]. To clarify the effects of TOFA treatment on T cells, we evaluated the numbers of CD4^+^ T cell subsets within the spleens of untreated BWF1 mice and mice treated with TOFA and/or DEXA. Total cell number was markedly decreased by treatment (data not shown) and it made difficult to compare each T cell subset statistically using cell number. Therefore, we compared each T cell subset based on the frequency. TOFA treatment resulted in significant increases in the frequency of splenic CD4^+^CD44^low^CD62L^high^ naïve/inactive T cells as compared with levels observed in control mice (Fig. 3a, left). Conversely, mice treated with TOFA exhibited a reduced frequency of CD4^+^CD44^high^CD62L^low^ effector/memory T cells (T_EM_) as compared with the control population; however, this decrease was only significant in the TOFA + DEXA therapy group (Fig. 3a, right). Additionally, there were significant decreases in the populations of both CD4^+^CD69^high^- and CD4^+^CD25^high^-activated T cells in the spleens of mice treated with TOFA alone or in combination with DEXA (Fig. 3b).

### CD4^+^CD25^high^Foxp3^+^ regulatory T cell (T_reg_) normalization in TOFA-treated mice

Of the effector T cell subsets, CD4^+^CD25^high^Foxp3^+^ T_reg_ cells are pivotal for maintaining peripheral self-tolerance and controlling autoimmunity by suppressing the activation and expansion of autoreactive T cells and other pathogenic immune cells [19]. In this study, similar to T_EM_ cells, we detected significant decreases in the populations of T_reg_ cells in TOFA-treated mice (Fig. 3c, left). Additionally, we detected significant reductions in the expression of *Foxp3*, the master regulatory gene of T_reg_ cells [19], within the kidney tissues of TOFA + DEXA-treated mice as compared to levels observed in controls (Fig. 3c, right).

### TOFA treatment modulates cytokine expression in BWF1 mouse kidneys and whole blood

**Fig. 4:**
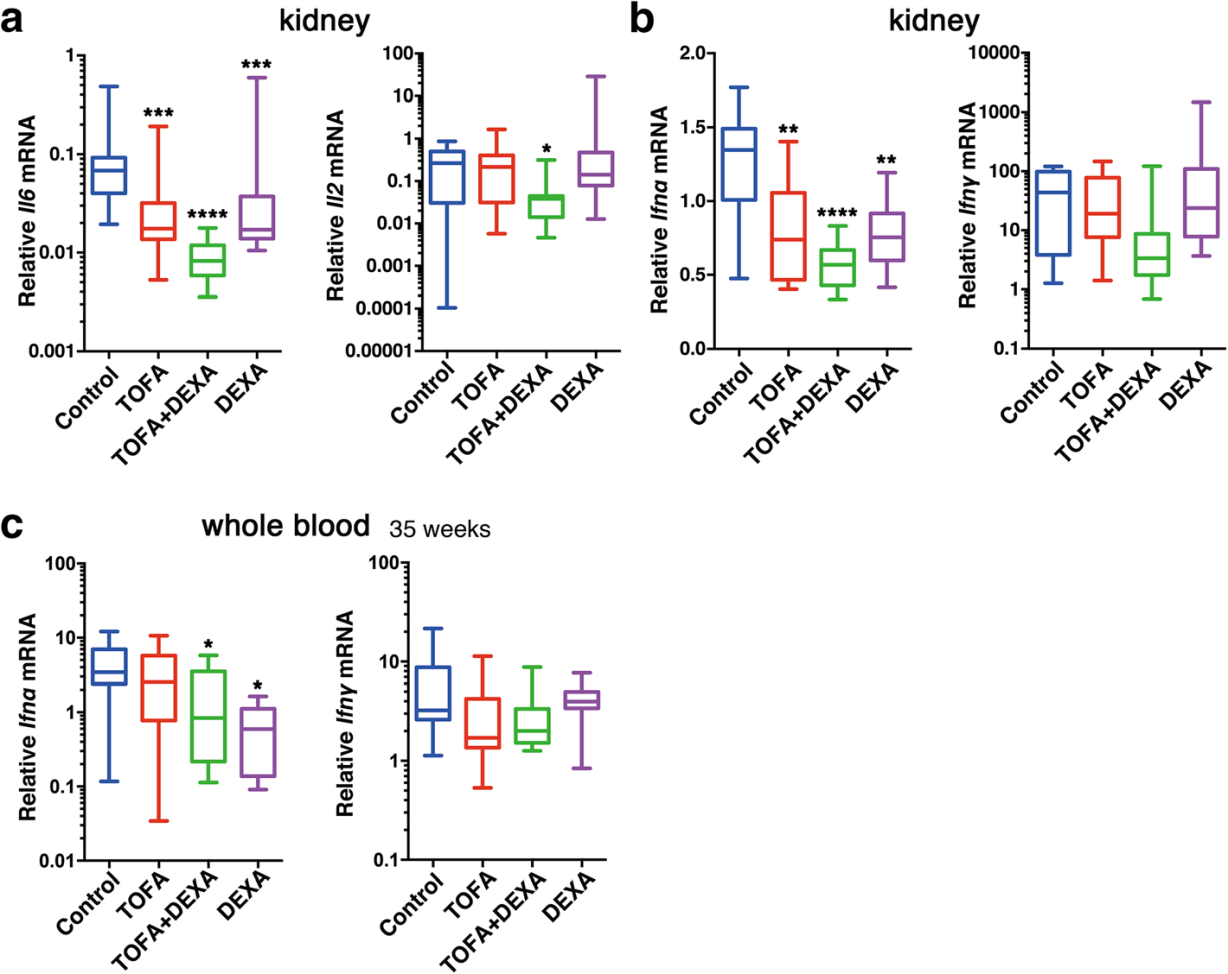
TOFA suppressed cytokine expression in kidneys and whole blood from BWF1. (**a**) *Il6*, *Il2*, (**b**) *Ifnα,* and *Ifnγ* expression in kidneys. (**c**) Total RNA extraction from whole-blood samples and evaluation of (**c**, left) *Ifnα* and (**c**, right) *Ifnγ* expression. Each represents [TOFA (*n* = 13); TOFA + DEXA (*n* = 14–15); DEXA (*n* = 7–8); and control (*n* = 11–13)]. *p < 0.05, **p < 0.01, ***p < 0.001 and ****p < 0.0001 (vs. control mice)

SLE is associated with increased expression of a wide variety of cytokines, with their expression linked to both pathogenesis and inflammation in SLE patients [18]. Therefore, we evaluated the expression of several pro-inflammatory cytokines in untreated and TOFA-treated mice. We detected the most significant decreases in *Il6* expression in kidney tissues of mice in the TOFA + DEXA groups (Fig. 4a, left). *Il2* expression also showed similar decreases, and significant decreased level in only TOFA + DEXA treatment group (Fig. 4a, right). We observed more significant decreases in *Ifnα* expression in kidney tissues of mice treated with TOFA and TOFA + DEXA (Fig. 4B, left), and there was decreased *Ifnγ* expression in kidneys of TOFA-treated mice; however, this decrease was not statistically significant (Fig. 4b, right). Similarly, there was a significant decrease in *Ifnα* expression in the blood of mice receiving dual TOFA + DEXA or single DEXA therapy at the age of 35 weeks (Fig. 4c, left). Additionally, whole blood *Ifnγ* expression was decreased in TOFA-treated groups, but those decreases were not significant (Fig. 4c, right).

### TOFA treatment suppressed IFN-induced gene *Ifit3*/*IFIT3* expression in CD4^+^ and CD3^+^ T cells from SLE mice and SLE patients, respectively

**Fig. 5:**
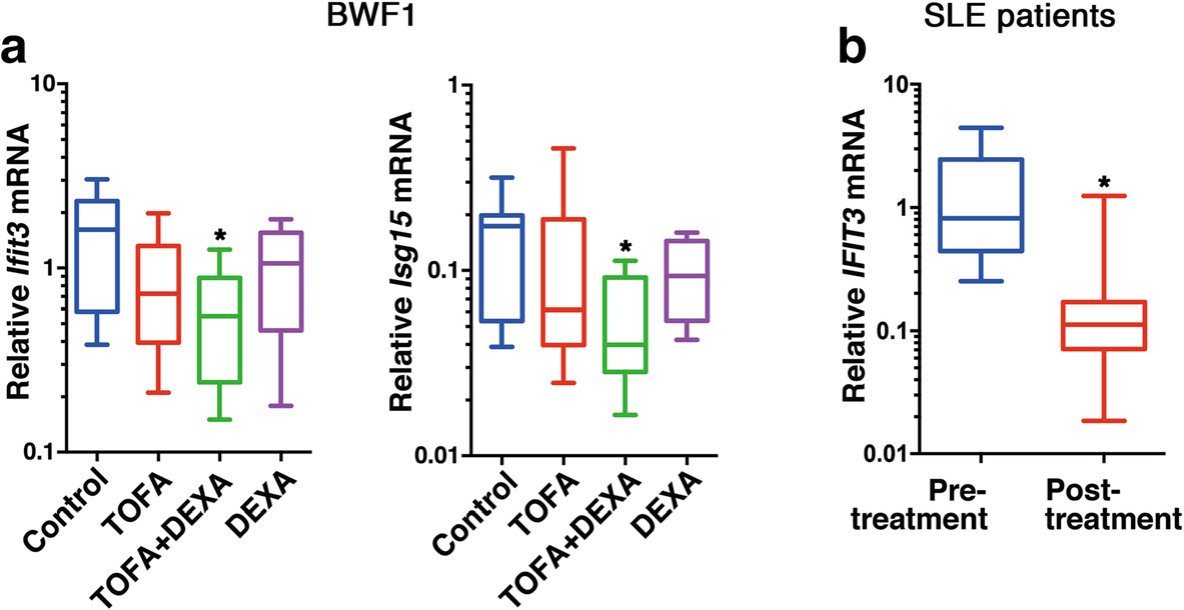
TOFA suppressed *Ifit3* and *Isg15* expression in BWF1 mice and *IFIT3* expression was also inhibited after immunosuppressive treatment. (**a**) *Ifit3* and *Isg15* expression associated with the IFN-signalling pathway in splenic CD4^+^ T cells from BWF1 mice. Each represents [TOFA (*n* = 8); TOFA + DEXA (*n* = 15); DEXA (*n* = 5); and control (*n* = 8)]. *p < 0.05 (vs. control mice). (**b**) *IFIT3* expression in each CD3^+^ T cell population from SLE patients (*n* = 9) pre- and post-immunosuppressive treatment including steroid. *p < 0.05 (vs. pre-treatment)

We investigated fluctuations in gene expression in CD4^+^ T cells isolated from spleens of SLE-prone mice and in CD3^+^ T cells from PBMCs of SLE patients. Initially, comparative-expression analyses were utilised to identify genes specifically inhibited by TOFA treatment in BWF1 mice. As described above, high levels of *Ifnα* expression were frequently detected in both the kidneys and whole blood of lupus-prone mice (Fig. 4b and c). Therefore, we re-analysed the expression profiles of comparative-expression analyses using IPA software, selecting genes associated with the IFN-signalling pathway from the IPA library. The expression of IFN-signalling-pathway related genes *Ifitm2*, *Ifitm3*, *Ifit3*, *Oas1a,* and *Isg15* appeared to be specifically reduced in TOFA-treated mice as compared with that observed in DEXA-treated mice (Additional file 2: Table S4). We subsequently confirmed the expression of these genes in the same CD4^+^ T cell RNA samples via qRT-PCR analysis. While we observed reduction of several gene expression following TOFA treatment in DNA microarray and the IPA analysis, this decrease was only significant for *Ifit3* and *Isg15* in qRT-PCR analysis (Fig. 5a).

We then analysed *IFIT3* expression in CD3^+^ T cells harvested from SLE patients between active pre- and inactive post-treatment phases. We detected a significant decrease in *IFIT3* expression following treatment (Fig. 5b). *ISG15* expression in CD3^+^ T cells harvested from SLE patients was also analysed and the alteration tended to decrease, however it was not significant (data not shown).

## Discussion

In this study, we demonstrated significant decreases in anti-dsDNA antibodies, proteinuria, and splenomegaly in TOFA alone and TOFA + DEXA-administered SLE-mouse group (Fig. 1) and amelioration of glomerular nephritis in TOFA + DEXA-treated SLE-prone mouse, BWF1 (Fig. 2). Previous report revealed that TOFA is immunologically effective for another SLE-prone mouse, MRL [20] with different genetic background from BWF1. TOFA + DEXA treatment was also clinically effective for MRL (Additional file 1: Figure S1). These data demonstrated that TOFA+ 0.5 mg/kg DEXA dual-therapy was clinically effective for treating SLE-prone mice, independent of genetic differences. Additionally, while 1 mg/kg DEXA is commonly used to treat SLE patients with severe nephritis or other severe organ complications [21], our findings suggested that TOFA administration might constitute a new therapeutic avenue and reduce the amount of steroids required to treat SLE and the complications.

We detected significant increases in the population of naïve/inactive T cells and significant decreases in T_EM_ cells in TOFA-treated SLE mice, indicating that TOFA was capable of suppressing abnormally activated lymphocytes, particularly T cells. Interestingly, a recent report also demonstrated that TOFA attenuates arthritis manifestations and reduces the pathogenic IFNγ^+^CD4^+^ splenic T cells in adjuvant arthritis rats [22]. In addition, we detected significant reductions in the frequency of T_reg_ cells and reduced *Foxp3* expression in kidney tissues of TOFA-treated mice (Fig. 3c, right). A previous report suggested that defects in T cell suppression observed in SLE patients are due to CD4^+^CD25^−^ effector-cell resistance and not abnormal CD4^+^CD25^+^Foxp3^+^ T_reg_ cell function [23], whereas a separate study demonstrated that T_reg_ cells from BWF1 mice are not predisposed to functional incompetence, but rather are present in a highly activated state [24]. Based on our findings and those of these studies, we concluded that TOFA and TOFA + DEXA treatment might result in reduced populations of activated effector T cells and T_reg_ cells to settle into a normal condition.

Exacerbation of SLE in BWF1 mice occurs in a type I IFN-dependent manner [25]. We detected reduced *Ifnα* expression in the kidneys and whole blood of TOFA-treated mice (Fig. 4b and c). The *IFN*-signature gene is associated with serological disease manifestation in SLE patients [26], and *IFN*-signature gene analysis using mRNA from whole blood was applied to SLE patients treated with an anti-IFN antibody [27, 28]. The analysis of mRNA from whole blood constituted a relatively simple approach to evaluate *Ifnα* and *Ifnγ* expression in BWF1 mice (Fig. 4c). Thus, whole blood *Ifn*/*IFN* analysis could prove a useful method for detecting serological SLE-disease manifestation and treatment effect in mice.

Our previous data, which were obtained by a recently developed DNA microarray, indicated that certain IRF-related genes (*IRF7*, *ISG15*, and *IFITM1*) in PBMCs demonstrate a significantly increased quantitative expression in the active SLE phase when compared with the genes from both the inactive phase of SLE and normal controls [2]. A recent genome-wide DNA-methylation study detected hypomethylation and overexpression of type I IFN-regulated genes, including *IFIT3* and *ISG15*, in naïve CD4^+^ T cells derived from SLE patients [29]. These data indicated that abnormal methylation exists in the T cells of individuals with lupus, even before activation. Furthermore, they provided evidence regarding the hyper-responsiveness of these T cells to type I IFN. In another report, significantly enhanced expression of five type I IFN-inducible genes, including *IFIT4* (same gene sequence as *IFIT3*), was detected in PBMCs from SLE patients as compared with levels detected in healthy and non-SLE controls [30]. Moreover, an analysis of T cells harvested from SLE patients suggested that *IFIT3* is more highly expressed in CD4^+^ T cells [31]. Meanwhile, ISG15 has been known as an activator of natural killer (NK) cells and a driver of IFNγ secretion [32]. In addition, ISG15 has emerged as a potentially critical bridge between type I and type II IFN immune response [33]. Here, TOFA + DEXA treatment led to a marked decrease in *Ifit3* and *Isg15* expression in the CD4^+^ T cells of BWF1 mice (Fig. 5a), whereas immunosuppressive treatment including steroid resulted in significant decreases of *IFIT3* expression in CD3^+^ T cells obtained from SLE patients (Fig. 5b). *ISG15* expression in CD3^+^ T cells also tended to decrease, but the alteration was not significant. Our data suggested that IFIT3 could get involved with SLE pathogenesis and modulate disease progression in both BWF1 mice and humans.

## Conclusions

The data presented here provided evidence that control of type I IFN signalling via JAK-STAT pathway following TOFA + DEXA and TOFA alone treatment improved disease conditions in BWF1 mice via down-regulation of cytokines, including IFN-α, and IFN-regulated genes expression. Especially, TOFA + DEXA treatment improved disease conditions in any analysis. TOFA treatment was also effective in alleviating SLE symptoms in MRL mice, indicating that this compound could comprise an effective anti-IFN and other cytokines therapy independent of pathogenic disease activity in SLE. Additionally, the therapeutic efficacy of TOFA treatment may allow for reduced steroid administration to lupus patients.

## Methods

### Mice

NZB/NZW F1 (BWF1) female mice and MRL/lpr (MRL) were purchased from Japan SLC, Inc. (Hamamatsu, Japan). The mice were maintained under clean pathogen-diminished conditions at the animal facilities of the Institutes for Environmental and Gender-specific Medicine, Juntendo University Graduate School of Medicine.

### Patients

Peripheral blood samples were obtained from nine female SLE patients ranging in age from 19 to 67 years. The patients were diagnosed with SLE according to the 1982 revised criteria of the American College of Rheumatology, and disease activity was evaluated on the basis of the SLE Disease Activity Index (SLEDAI). The patients were hospitalised for the onset or flare-up of their SLE and treated with corticosteroids / immunosuppressants.

### In vivo treatment

Age-matched female BWF1 mice (24-weeks old; *n* = 8–15 for each group) and MRL mice (ten-weeks old; *n* = 4–6 for each group) were randomised and ear tagged for identification, and initial baseline measurements, including body mass and serum samples, were collected prior to dosing. Six time per week, both mouse groups were orally administered 30 mg/kg tofacitinib (TOFA; CP-690,550; Pfizer, New York, NY, USA) diluted in saline. Dexamethasone (DEXA; Decadron; MSD K.K., Tokyo, Japan) was provided as the standard of care at 0.5 mg/kg via subcutaneous injection three times per week. Saline was utilised as the control and was administered orally six times per week or subcutaneously three times per week. All treatments were continued for 11 weeks in BWF1 mice and for 10 weeks in MRL. Both mice were sacrificed at 35- and 20-weeks old, respectively.

### Urine and antibody analysis

Sufficient spot urine was subjected to pyrogallol red molybdate method for measuring urine total proteins (Protein Assay Rapid Kit wako; Wako Pure Chemical Industries, Ltd., Osaka, Japan). Serum dsDNA-specific antibody levels were measured using a mouse anti-dsDNA antibody ELISA kit (AKRDD-061; Shibayagi Co., Ltd., Shibukawa, Japan).

### Histological analysis

Kidneys were harvested from mice, fixed in 20% formalin, embedded in paraffin, sectioned, haematoxylin and eosin (H&E), and periodic acid Schiff (PAS) staining. For immunofluorescence staining, kidneys were fixed in 4% paraformaldehyde at 4 °C and embedded in Tissue-Tek O.C.T. compound (Sakura Finetek, Tokyo, Japan) or directly embedded in compound and frozen at −80 °C. Fixed and unfixed tissues were then sectioned using a cryostat and stained with Alexa Fluor 488 goat anti-mouse IgG (H + L), Alexa Fluor 488 goat anti-mouse IgM (μ chain) (Invitrogen, Carlsbad, CA, USA), and rat anti-mouse C3 (Santa Cruz Biotechnology, Dallas, TX, USA), and with rat anti-mouse C4 (Santa Cruz Biotechnology) and rat anti-mouse C1q (Hycult Biotech, Uden, Netherlands). Meanwhile, H&E and PAS-stained sections were classified into six lupus-nephritis types based on the classification system of the 2003 International Society of Nephrology/Renal Pathology Society (ISN/RPS). The size (unit area) and mean fluorescence intensity (MFI) of the immunofluorescence staining in the digital images were measured by outlining the glomerulus with the polygon function of ImageJ Software (https://imagej.nih.gov/ij/). Finally, the severity of glomerular nephritis was decided by the glomerulus score based on the MFI of the immunofluorescence and the classification of ISN/RPS. The glomerulus score and cell-infiltration levels score are shown in Additional file 2: Table S1 and S2, respectively. Photomicrographs were obtained using a BZ-9000 microscope (Keyence, Osaka, Japan).

### Flow cytometry

To obtain single cell suspensions, spleens from mice in the control and treatment groups were crushed and passed through 40 μm pore nylon mesh. The resulting cell suspensions were then stained at 4 °C in PBS containing 0.1% BSA (Wako) and 0.09% NaN_3_ (Wako) using antibodies specific to the following mouse proteins: CD4 (clone GK1.5), CD44 (clone IM7), CD62L (clone MEL-14), TCRβ (clone H57–597), CD69 (clone H1.2F3), CD25 (clone PC61) and Foxp3 (clone MF23). All antibodies were obtained from BioLegend (San Diego, CA, USA) for BD Biosciences (San Jose, CA, USA). Foxp3 staining was performed using the Foxp3 staining kit (eBioscience, San Diego, CA, USA), according to the manufacturer’s protocol. Cells were analyzed using a FACSCalibur system (BD Biosciences), and the data were analyzed using FlowJo software (Tree Star, Ashland, OR, USA).

### DNA microarray and data analysis

CD4^+^ and CD3^+^ T cells were isolated from mouse spleens and peripheral blood mononuclear cells (PBMCs) harvested from human patients, using a mouse CD4^+^ cell-isolation kit and human CD3^+^ MicroBeads (Miltenyi Biotec, Bergisch Gladbach, Germany), respectively, followed by an autoMACS separator device (Miltenyi Biotec). RNA extraction was performed as described in the following section. DNA microarray methods were the same as our previous report [8]. The obtained numerical data were normalised per slide and per channel to the expression level of the housekeeping gene *Atp5f1*. Genes with MFIs ≤100 as compared to the background and based on the results of dye-swapping experiments were considered negatively expressed. Genes found to be differentially expressed in the treated versus untreated SLE-mouse samples according to the microarray analysis results were functionally categorised, and their interactions were clarified using Ingenuity Pathway Analysis (IPA) software (Ingenuity Systems, Redwood City, CA, USA).

### RNA amplification and quantitative RT-PCR (qRT-PCR)

RNA was extracted from mouse whole blood and kidney samples using RNAiso Blood (TaKaRa Bio) and ISOGEN II (Nippon Gene Co., Ltd., Toyama, Japan) kits, respectively, and from mouse CD4^+^ (in spleen) and human CD3^+^ T cells (in PBMCs) using an RNeasy mini kit (Qiagen, Venlo, Netherlands). The methods of quantification of mRNA expression levels and qRT-PCR analysis of CD3^+^ T cell-derived samples were the same as our previous report [8]. The expression levels of mouse *Actb* and human *TFRC* were used as internal controls for the CD4^+^ and CD3^+^ T cell samples, respectively. All primers are shown in Additional file 2: Table S3.

### Statistical analyses

Microarray and qRT-PCR data were analysed using Microsoft Excel (Microsoft, Redmond, WA, USA), GraphPad InStat software package 6.0 (GraphPad Software, La Jolla, CA, USA) and the statistical package R (version 3.3.2; available as a free down-load from http://www.r-project.org), respectively. Unpaired *t* tests, two-way factorial analysis of variance tests, and non-parametric Kruskal-Wallis tests were applied for comparisons of data from treated versus untreated groups and from BWF1 and MRL mice receiving TOFA/TOFA + DEXA versus DEXA treatment, respectively. A *p* < 0.05 was considered statistically significant for all tests.

## Additional files

Additional file 1: Figure S1. TOFA ameliorated splenomegaly and interstitial nephritis in MRL mice Spleen size **(a, left)** and weight in MRL **(a, right)**. Boxplot shows median and interquartile ranges from minimum to maximum. **(b, c)** MRL were evaluated for interstitial nephritis. **(b)** Representative H&E stained kidney sections in control and treatment groups. Arrows: areas of cell infiltration. **(c)** Levels of cell infiltration in each treatment group (mean ± SD). Each graph represents [TOFA (*n* = 6); TOFA + DEXA (*n* = 5); DEXA (*n* = 4); and control (*n* = 6)]. *p < 0.05; **p < 0.01 and ***p < 0.001 (vs. control mice). DEXA: dexamethasone; MRL: MRL/lpr mice; TOFA: tofacitinib. (JPG 2682 kb)

Additional file 2: Table S1. The glomerulus score. **Table S2.** The score of cell-infiltration level. **Table S3.** The primers used for this study. **Table S4.** Expression levels of genes associated with the interferon (IFN) signaling pathway in splenic CD4^+^ T cells from BWF1 mice. (DOCX 27 kb)

## Abbreviations

IFIT3: interferon-induced protein with tetratricopeptide repeats 3
IFITM: interferon-induced transmembrane
IFN: interferon
ISG15: Interferon-stimulated gene 15
JAK: Janus kinase
Oas1a: 2′-5′ oligoadenylate synthetase 1a
SLE: systemic lupus erythematosus
STAT: signal transducers and activator of transcription
