# Supplementary figures and images for: JAK inhibitor has the amelioration effect in lupus-prone mice: the involvement of IFN signature gene downregulation
Source: BMC Immunol. 2017 Aug 22;18:41. doi: 10.1186/s12865-017-0225-9 (PMC5568047; doi:10.1186/s12865-017-0225-9)

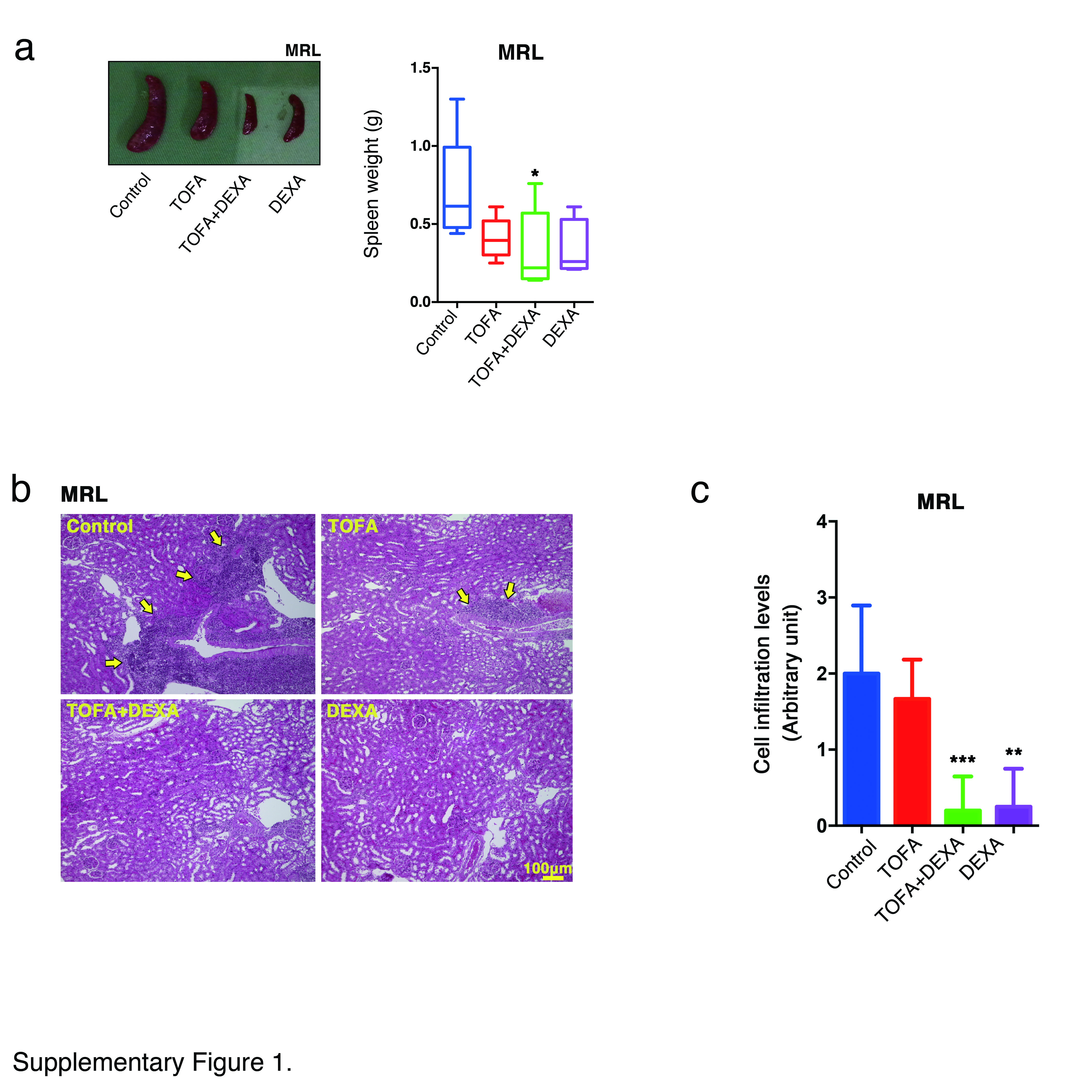

Supplement: Supplementary file 1 — TOFA ameliorated splenomegaly and interstitial nephritis in MRL mice Spleen size (a, left) and weight in MRL (a, right). Boxplot shows median and interquartile ranges from minimum to maximum. (b, c) MRL were evaluated for interstitial nephritis. (b) Representative H&E stained kidney sections in control and treatment groups. Arrows: areas of cell infiltration. (c) Levels of cell infiltration in each treatment group (mean ± SD). Each graph represents [TOFA (n = 6); TOFA + DEXA (n = 5); DEXA (n = 4); and control (n = 6)]. *p < 0.05; **p < 0.01 and ***p < 0.001 (vs. control mice). DEXA: dexamethasone; MRL: MRL/lpr mice; TOFA: tofacitinib. (JPG 2682 kb) [file 12865_2017_225_MOESM1_ESM.jpg]
