# Supplementary material for: JAK inhibitor has the amelioration effect in lupus-prone mice: the involvement of IFN signature gene downregulation
Source: BMC Immunol. 2017 Aug 22;18:41. doi: 10.1186/s12865-017-0225-9 (PMC5568047; doi:10.1186/s12865-017-0225-9)
Supplement: Supplementary file 2 — The glomerulus score. Table S2. The score of cell-infiltration level. Table S3. The primers used for this study. Table S4. Expression levels of genes associated with the interferon (IFN) signaling pathway in splenic CD4+ T cells from BWF1 mice. (DOCX 27 kb) [file 12865_2017_225_MOESM2_ESM.docx]

**Table S1.** The glomerulus score.

In an x200-magnified image of kidney section from BWF1 mice, the glomerulus that admits the following items is counted and the number is defined as the glomerulus score (0 to 36). The evaluation of glomerular lesions is based on the classification system of the 2003 International Society of Nephrology / Renal Pathology Society.

| Methods of observation | Items | | Score |
| --- | --- | --- | --- |
| Light Scope  of HE and PAS staining | Glomerular lesions | Normal | 0 |
|  |  | Inconsequential lesion | 1 |
|  |  | Focal and Segmental | 2 |
|  |  | Focal and Global | 3 |
|  |  | Diffuse and Segmental | 4 |
|  |  | Diffuse and Global | 5 |
|  | Mesangial expansion | Normal | 0 |
|  |  | Mild | 1 |
|  |  | Moderate | 2 |
|  |  | Advance | 3 |
|  | Crescent formation | 0 / 20 Glomerulus | 0 |
|  |  | 1 to 5 / 20 | 1 |
|  |  | 6 to 10 / 20 | 2 |
|  |  | 11 to 15 / 20 | 3 |
|  |  | 16 to 20 / 20 | 4 |
|  | Glomerular sclerosis | 0 / 20 Glomerulus | 0 |
|  |  | 1 to 5 / 20 | 1 |
|  |  | 6 to 10 / 20 | 2 |
|  |  | 11 to 15 / 20 | 3 |
|  |  | 16 to 20 / 20 | 4 |
| Immunofluorescence  of IgG, IgM, C3, C4 and C1q | Glomerular fluorescence intensity | 0 / 20 Glomerulus | 0 |
|  |  | 1 to 5 / 20 | 1 |
|  |  | 6 to 10 / 20 | 2 |
|  |  | 11 to 15 / 20 | 3 |
|  |  | 16 to 20 / 20 | 4 |

**Table S2.** The score of cell-infiltration level.

In an x100-magnification image of kidney section from MRL mice, the cell-infiltration level was scored (0 to 3) by measuring the area of cell infiltration within the renal interstitial area.

| Score | The area of cell infiltration within the renal interstitium |
| --- | --- |
| 0 | No infiltration |
| 1 | Moderate infiltration |
| 2 | Intermediate levels of infiltration (between scores of 1 and 3) |
| 3 | >25% of the tissue exhibits infiltration |

**Table S3.** The primers used for this study.

| Gene Symbol | Forward | Reverse |
| --- | --- | --- |
| **Murine** |  |  |
| *Actβ* | 5’-CATCCGTAAAGACCTCTATGCCAAC-3’ | 5’-ATGGAGCCACCGATCCACA-3’ |
| *Il6* | 5’-CCACTTCACAAGTCGGAGGCTTA-3’ | 5’-GCAAGTGCATCATCGTTGTTCATAC-3’ |
| *Il2* | 5’-GCTGTTGATGGACCTACAGGA-3’ | 5’-TTCAATTCTGTGGCCTGCTT-3’ |
| *Foxp3* | 5’-ATCTGTGGCCTCAATGGACAAG-3’ | 5’-AGTAGTCCATGTTGTGGAAGAACTC-3’ |
| *Ifnα* | 5’-CATTCTGCAATGACCTCCAC-3’ | 5’-TCAGGGGAAATTCCTGCAC-3’ |
| *Ifnγ* | 5’-CGGCACAGTCATTGAAAGCCTA-3’ | 5’-GTTGCTGATGGCCTGATTGTC-3’ |
| *Ifitm2* | 5’-CTCAGCTGTTGTGAGGACCA-3’ | 5’-CCACCATCTTCCTGTCCCTA-3’ |
| *Ifitm3* | 5’-AACATGCCCAGAGAGGTGTC-3’ | 5’-CTTAGCAGTGGAGGCGTAGG-3’ |
| *Ifit3* | 5’-GAGGACAACCGGAAGTGTGT-3’ | 5’-GGATGAGCAGAGGAGTCAGG-3’ |
| *Oas1a* | 5’-CTGTCCACCTGTTGGAAGGT-3’ | 5’-GGCTTTGCTCTAACCACTGC-3’ |
| *Isg15* | 5’-ACCCTTTCCAGTCTGGGTCT-3’ | 5’-AGCCAGAACTGGTCTTCGTG-3’ |
| **HUMAN** |  |  |
| *TFRC* | 5’-GCGAGCACTGACCAGATAAGAATG-3’ | 5’-TCCCGATAATGTGTTAGGATTGTGA-3’ |
| *IFIT3* | 5’-CTGAACTTGACTGTGAGGAAGG-3’ | 5’-TGGGTTGTTGGGCTTTTC-3’ |
| *ISG15* | 5’-ACCCTTTCCAGTCTGGGTCT-3’ | 5’-AGCCAGAACTGGTCTTCGTG-3’ |

**Table S4.** Expression levels of genes associated with the interferon (IFN) signaling pathway in splenic CD4^+^ T cells from BWF1 mice.

| Gene  Symbol | Gene Bank ID | Gene Name | Gene Expression Ratio | | |
| --- | --- | --- | --- | --- | --- |
|  |  |  | **TOFA** | **TOFA**  **+DEXA** | **DEXA** |
| ***Ifitm2*** | NM_030694 | interferon induced transmembrane protein 2 | 0.92 | 0.46 | 0.51 |
| ***Ifitm3*** | NM_025378 | interferon induced transmembrane protein 3 | 0.72 | 0.47 | 0.58 |
| ***Ifit3*** | NM_010501 | interferon-induced protein with tetratricopeptide repeats 3 | 0.73 | 0.41 | 0.90 |
| ***Oas1a*** | NM_145211 | 2'-5' oligoadenylate synthetase 1A | 0.56 | 0.36 | 0.45 |
| ***Isg15*** | NM_015783 | interferon stimulated gene 15 | 0.60 | 0.41 | 0.56 |

TOFA, tofacitinib; DEXA, dexamethasone
